# Predicted efficacy and tolerance of different dosage regimens of benzylpenicillin in horses based on a pharmacokinetic study with three IM formulations and one IV formulation

**DOI:** 10.3389/fvets.2024.1409266

**Published:** 2024-05-31

**Authors:** Aude A. Ferran, Béatrice B. Roques, Laura Chapuis, Taisuke Kuroda, Marlène Z. Lacroix, Pierre-Louis Toutain, Alain Bousquet-Melou, Elodie A. Lallemand

**Affiliations:** ^1^INTHERES, Université de Toulouse, INRAE, ENVT, Toulouse, France; ^2^Clinical Veterinary Medicine Division, Equine Research Institute, Japan Racing Association, Shimotsuke, Japan; ^3^Department of Comparative Biomedical Sciences, The Royal Veterinary College, University of London, London, United Kingdom

**Keywords:** benzylpenicillin, penicillin, horse, PK/PD, pharmacokinetics, tolerance

## Abstract

**Introduction:**

Benzylpenicillin (BP) is a first-line antibiotic in horses but there are discrepancies between manufacturers and literature recommendations regarding dosing regimen. Objectives of this study were to evaluate pharmacokinetics and local tolerance of four different formulations of BP in adult horses, and to suggest optimized dosing regimen according to the formulation.

**Methods:**

A cross-over design was used in 3 phases for the intramuscular injection of three different products: procaine BP alone, procaine BP/ benzathine BP combination or penethamate hydriodide were administered IM in the gluteal muscles of 6 horses for 3 days. Single IV administration of sodium BP was performed to the same horses with a dose of 22,000 IU BP/kg bwt 39 weeks after last IM injection. BP plasma concentrations were determined by UPLC assay coupled with mass spectrometry and a PK/PD analysis was conducted to predict the efficacy of various dosing regimens by estimating values of the fT>MIC index for different minimum inhibitory concentrations (MIC). Tolerance at the site of IM injection was monitored by creatine kinase activity quantified with a validated chemistry system and clinical scorings.

**Results and discussion:**

Except one neurological reaction following one administration of penethamate hydriodide, the tolerance was good. Procaine BP alone, procaine BP/benzathine BP combination or penethamate hydriodide intramuscular administrations at a dosage of 22,000 IU BP/kg bwt q24h for 5 days would yield plasma concentrations that should be effective against bacteria with MIC of ≤0.256, 0.125 or 0.064 mg/L respectively. Of all the tested treatments, the use of a sodium BP by IV Constant Rate Infusion (CRI) for 10 hours a day was deemed to be the most efficient. All the formulations tested in this study are adequate to treat infections with susceptible *Streptococcus equi*.

## 1 Introduction

Facing the global issue of antibiotic resistance, it is of paramount importance to optimize dosing regimen of first-line antibiotic treatments in order to maintain their efficacy and limit the use of second-line antibiotics that are more critical for human health. In horses, benzylpenicillin (BP),which was administered as procaine BP, sodium BP, potassium BP, benzathine BP, or penethamate hydriodide, is a first-line antibiotic (1). It is reported as the most frequently used beta-lactam drug in equine medicine in the EU (2) and is classified in category D by the European Medicines Agency, i.e., the category with the lower impact for human health (3). BP is recommended in the treatment of infections caused by gram-positive aerobic pathogens such as *Streptococcus* spp. and susceptible *Staphylococcus aureus* and gram-positive anaerobic organisms with the exception of *Bacteroides fragilis*. Some gram-negative bacteria such as many *Pasteurella* spp. are also susceptible to BP (4). The use of intravenous (IV) sodium BP and intramuscular (IM) procaine BP is very common, and pharmacokinetics (PK) of those formulations have already been studied (5–9); but discrepancies between manufacturer’s and literature recommendations do exist for dosing regimens of procaine BP by IM route. Indeed, a manufacturer recommends doses ranging from 12,460 to 30,260 IU/kg q24h that correspond to daily doses of at least 30% lower than the one of 22,000 IU/kg q12h recommended in the literature (10, 11). In addition to procaine BP, penethamate hydriodide, a prodrug of BP, and a combination of procaine BP/benzathine BP are also authorized for the same indications in horses. Some practitioners use procaine BP/benzathine BP combination every other day or once a day instead of procaine BP twice a day. Others also switch from procaine BP to penethamate hydriodide (not containing procaine) for doping issue and the more convenient reduced volumes which were injected on days 2 and 3, according to the Summary of Product Characteristics. Despite these quite common uses, there are doubts on the efficacy of these formulations in horses (12), and to the best of our knowledge, there are no available PK data for penethamate hydriodide in horses, and only one old PK study in 1983 described the fate of benzathine BP in horses (6).

The objectives of our study were to determine tolerance at injection site and PK parameters in horses of four different available formulations of BP: sodium BP administered by intravenous route, and procaine BP alone, procaine BP/benzathine BP combination, and penethamate hydriodide alone administered by IM route. Another objective of this study was to predict the ability of these formulations of BP to cure infections using pharmacokinetic/pharmacodynamic (PK/PD) indices as a surrogate of clinical efficacy and suggest optimized dosing regimen according to the formulation.

## 2 Materials and methods

### 2.1 Animals

Six adult horses (3 mares and 3 geldings; 3 French trotters and 3 mixed breeds; mean age ± SD: 8.3 ± 2.9 years; mean weight ± SD: 503 ± 40 kg) were included. All horses were healthy based on a full physical examination. None of the horses had received antibiotic treatment within 6 months before the study. Horses were housed individually, had free access to water, and were fed maintenance diet. This study follows national and institutional guidelines for humane animal treatment and comply with relevant legislation in France. The study was approved by the Local and National Institutional Animal Care and Use Committees (APAFIS #19371-2019022112206504v2).

### 2.2 Study design

The 6 horses were first enrolled in a 2x2x2 crossover design with a washout of 4 to 5 months between two periods to compare three IM formulations: procaine BP alone, procaine BP/benzathine BP combination, and penethamate hydriodide alone. Horses 1 and 2 received procaine BP in phase 1, penethamate hydriodide in phase 2, and procaine BP/benzathine BP combination in phase 3. Horses 3 and 4 received procaine BP/benzathine BP combination in phase 1, procaine BP in phase 2, and penethamate hydriodide in phase 3. Horses 5 and 6 received penethamate hydriodide in phase 1, procaine BP/benzathine BP combination in phase 2, and procaine BP in phase 3. All 6 horses received IV administration in the same week. IM administrations were performed with total volume injected in one point in the gluteal muscles by the same veterinarian. Right side was used for the first injection, and left side was used for the second injection and right side for third injection if any. After completion of the crossover and a washout of 39 weeks, the same six horses received a single slow IV bolus over 2 min of sodium BP using a 13G catheter placed in the right jugular vein. Dosing regimen were chosen according to manufacturer’s recommendations for horses in the Summary of Product Characteristics (SPC) except for sodium BP which is a formulation marketed for humans. When doses were reported in mass (mg/kg bwt), they were harmonized in terms of IU of BP (IU/kg bwt) considering that 1 mg of BP is equivalent to 1780 IU of the second international standard reference. Details on registered name, administered doses, and injected volumes for the different formulations are shown in Table 1.

**Table 1:** Dosage regimen and intervals used for the different pharmaceutical products in the PK study on six horses.

Active ingredients	Trade name Pharmaceutical form [company]	Route of administration	SPC Dose in IU of BP	Dosing regimen used in the study in IU of BP	Injected volume*
Procaine BP	Dépocilline® Suspension [Intervet/MSD]	IM	12,460–30,260 IU/kg bwt q24h	17,800 IU/kg bwt q24h for 3 days	29.4 mL
Procaine BP + benzathine BP	Duplocilline® Suspension [Intervet/MSD]	IM	22,072 IU/kg bwt (unique administration)	22,072 IU/kg bwt q48h (2 administrations)	35.0 mL
Penethamate (as hydriodide)	Penetavet® Suspension [Boehringer Ingelheim]	IM	7.72 g *in toto* as penethamate at day1 followed by 3.86 g *in toto* at day2 and day3. In BP for a 500 kg horse: 21,205 IU/kg bwt at day1 followed by 10,603 IU/kg bwt at day 2 and day3.	10,6 millions IU *in toto* at Day 1 followed by 5,3 millions IU *in toto* at day 2 and day 3 In BP for a 500 kg horse: 21,205 IU/kg bwt at day1 followed by 10,603 IU/kg bwt at day 2 and day3.	30 mL day1 15 mL day 2 and day3
Sodium BP	Pénicilline G Panpharma 5MIU® Solution [Panpharma]	IV	22,000 IU/kg bwt q6h (literature recommendation)	22,000 IU/kg bwt Single dose	26.4 mL

* Injected volume per administration for a 500-kg horse. BP, benzylpenicillin; SPC, summary of product characteristics.

Blood samples (4 mL) were collected from jugular vein in heparinized tubes. Time 0 was defined as the end of the antibiotic injection. For IM administration, blood was collected prior to antibiotic injection at 0.25, 0.5, 1, 2, 4, 6, 8, 12, and 23.5 h post-injection at day 1, 0.5, 1, 2, 12, and 23.5 h at days 2 and 3; and once at days 4, 5, and 6. For the IV administration, blood was collected prior to antibiotic administration at 1, 5, 10, 15, and 30 min and at 1, 2, 3, 4, 6, 8, and 24 h after the end of the administration.

### 2.3 Tolerance

Clinical examinations were performed twice daily from day 1 to day 3, once a day from day 4 to day 6, and once a week for 3 weeks. Additionally, each horse was closely monitored 1 h after each injection to notice any adverse reaction.

Injection sites were monitored using a swelling score and an induration score (each from 0 to 3, 3 being the worst, Supplementary Tables S1, S2). A global pain score including vital parameters and lameness score was also calculated (from 0 to 21, 21 being the worst, Supplementary Table S3). Scores were attributed by evaluators who were blinded of treatment allocation. Local muscle loss was estimated after the quantification of plasmatic creatine kinase (CK) at 0, 1, 6, 12, and 23.5 h after first injection (13). The following equation was used: Q_muscle loss_ (g/kg bwt) = (AUC_IM_-AUC_IV_)x3.9 10^−6^ with Area Under the Curves in U.h/L.

### 2.4 Analysis of BP and CK concentrations

Samples of blood for BP concentration analysis were stored on melting ice and centrifuged within 1 h of collection at 3,000 g for 10 min. The supernatant was then stored at −80°C until analysis. BP concentrations in plasma were measured by Ultra High Performance Liquid Chromatography (Acquity UPLC, Waters) coupled to Xevo Triple Quadrupole Mass Spectrometer (Waters, MA, United States), as described previously (14). In brief, the method was validated with a calibration curve ranging from 0.01 to 10 μg/mL with a quadratic model weighted by 1/X^2^ (X = BP concentration). The coefficient of variation (CV %) of the intra-day and inter-day precisions was lower than 9% for both, with an accuracy varying from 91 to 108%. The limit of quantification (LOQ) was 0.01 μg/mL with a precision of 16% and an accuracy of 114%. Ampicillin was used as the internal standard.

Creatine kinase was quantified with a validated Vitros 350 automate (Ortho Clinical Diagnostics, Raritan, USA).

### 2.5 Statistical analysis

Statistical analyses were performed using RStudio (R 4.1.0, R Development Core Team, R Foundation for Statistical Computing, Vienna, Austria). Data normality was checked with a Shapiro–Wilk test and variance homogeneity with a Bartlett test if data normality was verified and with a Fligner test if this was not the case. A parametric one-way analysis of variance (ANOVA) was performed to test the influence of treatment on the area under the curve (AUC) for the plasma CK concentration-time curve and the swelling and induration score-time curve. A Bonferroni correction was applied for pairwise t-test. The differences were considered as statistically significant if *p* < 0.05.

### 2.6 Pharmacokinetic (PK) analysis

BP concentrations over time were analyzed using non-linear mixed-effects models (Monolix2023R1, Lixoft), to determine the PK parameters of BP with the different formulations and measure the variability between individuals. In our study, the precision of the parameters describing the absorption after IM route was increased by the fact that the same individuals received the same drug (BP) by IV and three IM formulations separated by wash-out periods.

Experimental data were best described by a two-compartmental model with (IM route) or without (IV route) a rate constant of absorption and a bioavailability factor. The error model error was a combined additive plus multiplicative one. As the elimination and distribution processes of BP were considered as independent of the route, unique values of V1 (volume of the central compartment), Q (distributional clearance between central and peripheral compartment), V2 (volume of the peripheral compartment), and Cl (clearance) were estimated for a given horse for the three IM formulations, while different ka (rate constant of absorption) and F (bioavailability) were estimated for each IM formulation. All parameters were assumed to follow a lognormal distribution in the population of horses except F for which a logit-normal distribution was assumed to prevent estimate F higher than 1 (100%). Modeling was performed with all the concentration data to estimate all PK parameters for both the population and each individual for the different tested formulations.

Evaluation of goodness-of-fit was based on the following plots: (i) individual and population predicted concentrations versus observed concentrations, (ii) individual plots (predicted observed vs. time), (iii) distribution of weighted residuals versus time and concentrations, (iv) distribution of the standardized random effects, (iv) probability of normalized prediction distribution errors (NPDE).

### 2.7 Pharmacokinetic–pharmacodynamic indices

By considering dose-linearity, the estimated PK parameters and their estimated variabilities among the individuals of a population were used to simulate with Simulx® (Lixoft, version 2023R1) the BP plasma concentrations that would be obtained in 500 horses receiving the different BP formulations with different simulated dosing regimens. For BP, a time-dependent antimicrobial, the efficacy can be predicted by using the PK/PD index *fT > MIC* that corresponds to the time (T) that the free plasma concentration of BP exceeds the Minimal Inhibitory Concentration (MIC) of the pathogen (15). In horses, the mean plasma protein binding of BP was reported to be 62.8+/−1.8% (16), and in our simulations, free fraction of unbound BP was considered equal to 40%. Efficacy of BP was assumed when free plasma concentrations were above a given MIC for 40% of the dosing interval (17) for 5 days of treatment. The percentages of target attainment (PTA), i.e., the percentages of horses from the simulated population achieving the targeted *fT > MIC* of 40% were calculated for different formulations, dosing regimens, and MIC.

## 3 Results

The results of the clinical examinations were unremarkable. One mare presented neurologic signs few minutes after the first injection of penethamate hydriodide during the first period (first known injection of BP). She was startled, then tensed, with muscle fasciculations, restless, and fearful. She slowly recovered within 5 min without any intervention. She did not present cardiorespiratory signs. Experiment was continued, and no further adverse reaction was noted after the subsequent administration of BP included in this formulation or other formulations.

### 3.1 Pharmacokinetics

**Figure 1:**
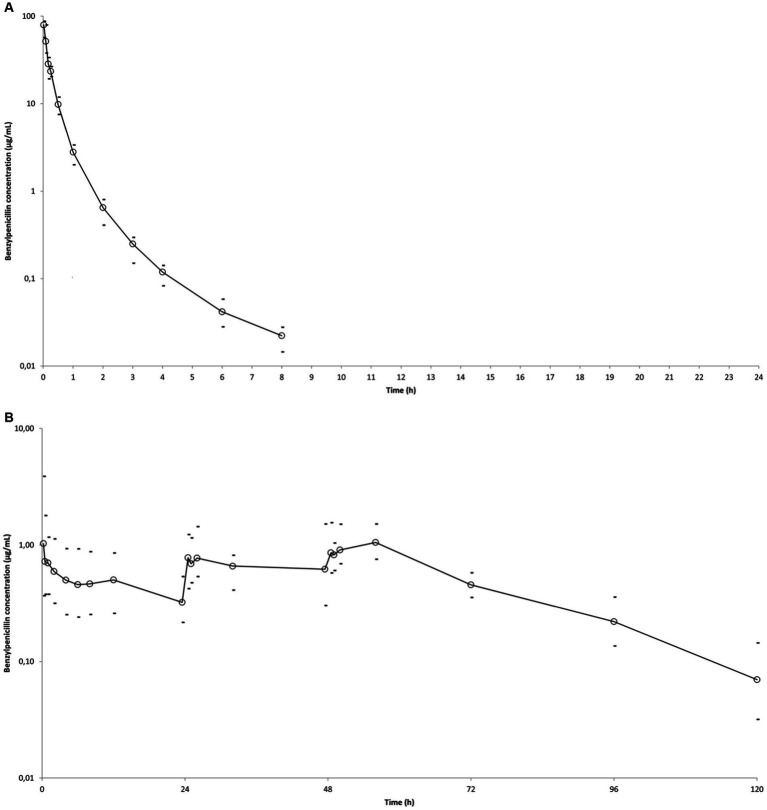

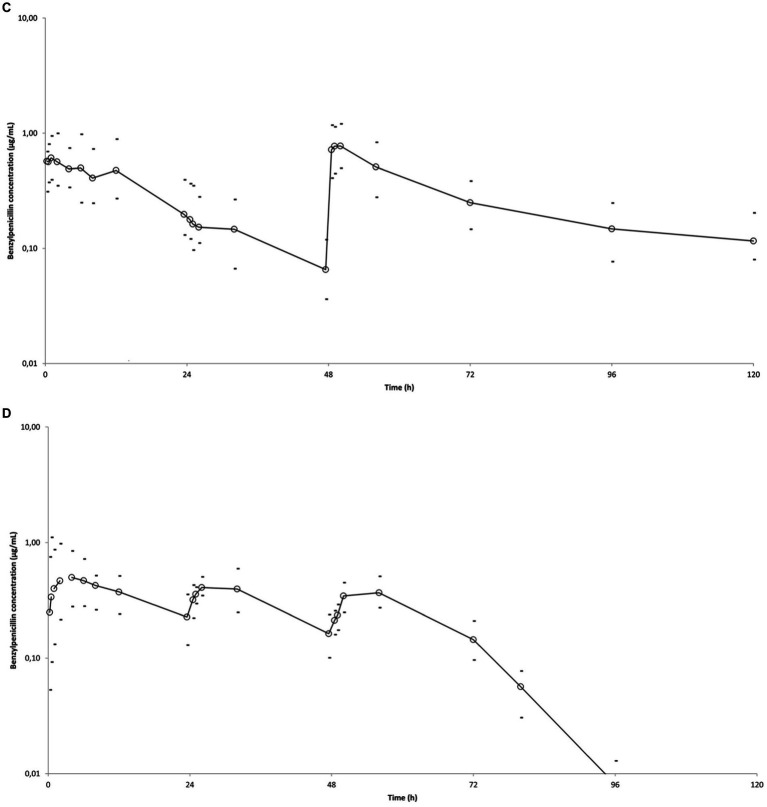
Mean observed BP plasma concentration (○) versus time curves in six horses after administrations of different BP formulations. Minimum and maximum individual values are indicated (−). **(A)** Single administration of sodium BP IV (22,000 IU/kg bwt) via a catheter in the right jugular vein. **(B)** IM administration of procaine BP alone (17,800 IU/kg BP bwt) at 0, 24, and 48 h. **(C)** IM administration of procaine BP/benzathine BP combination (22,072 IU/kg BP bwt) at 0 and 48 h. D: IM administration of penethamate hydriodide at 0 (10.6 M IU BP), 24 (5.3 M IU BP), and 48 h (5.3 M IU BP). Left axis is from 0.01 to 100 for **(A)** and from 0.01 to 10 for **(B–D)**, considering respective BP values obtained with the different formulations.

Plasma concentrations versus time profiles of BP are shown in Figure 1. Estimated population pharmacokinetic parameters and their between-subject variability among horses expressed as coefficients of variation are shown in Table 2. Bioavailabilities were 100% (CV = 4%) for procaine BP alone, 60% (CV = 37%) for procaine BP/benzathine BP combination, and 48% (CV = 18%) for penethamate hydriodide. BP clearance was 0.49 L/kg.h (CV = 21%), and the volume of the central compartment was 0.096 L/kg (CV = 48%).

**Table 2:** Estimated population PK parameters of BP [estimate and standard error (SE)] in horses including parameters of absorption [absorption rate (ka) and bioavailability (F)] for different formulations.

Parameters	Estimate	Units	SE	BSV (CV%)
Plasma clearance	0.49	L/h/kg	0.045	20.6
V1	0.096	L/kg	0.021	47.7
V2	0.071	L/kg	0.012	2.5
F_proc	1		0.0008	4.2
F_proc_benz	0.60		0.13	37.4
F_pene	0.48		0.047	18.1
Ka_proc	0.035	1/h	0.0057	38.2
Ka_proc_benz	0.048	1/h	0.0059	21.0
Ka_pene	0.043	1/h	0.0074	38.9

The coefficient of variation (CV%) describing the between-subject variability (BSV) among the individuals is also reported.

V1 (volume of the central compartment), V2 (volume of the peripheral compartment), Cl (plasma clearance) ka (rate of absorption), F (bioavailability); proc, procaine BP alone; proc-benz, procaine BP/benzathine BP combination; pene, penethamate hydriodide.

### 3.2 Pharmacokinetic–pharmacodynamic indices

Several BP PK profiles were simulated for the different dosing regimens with the different formulations for 5 days. The PTA with different formulations of BP and different dosing regimens for different MIC values from 0.016 to 1 μg/mL are shown in Table 3.

**Table 3:** Predicted PTA (percentage of target attainment, i.e., percentages of horses for which the target is reached) with different formulations of BP and different simulated dosing regimens for different MIC values from 0.016 to 1 μg/mL.

		MIC values (μg/mL)						
	Total dose/day (IU/day)	0.016	0.032	0.064	0.125	0.25	0.5	1
**Intramuscular administration simulated regimens**								
Procaine BP IM q12h	44,000	100	100	100	100	100	83	12
Procaine BP IM q24h	22,000	100	100	100	100	100	16	0
Procaine+benzathine BP IM q24h	22,000	100	100	100	96	17	0	0
Procaine+benzathine BP IM q48h	11,000	100	100	100	46	4	0	0
Penethamate IM q24h	22,000	100	100	100	88	0	0	0
**Intravenous administration simulated regimens**								
Bolus Sodium BP IV q8h	66,000	100	79	29	0	0	0	0
Bolus Sodium BP IV q6h	88,000	100	100	100	50	4	0	0
CRI Sodium BP IV over 10 h 2,200 IU/kg bwt/h for 10 h	22,000	100	100	100	100	100	50	8
CRI Sodium BP IV over 10 h 4,400 IU/kg bwt/h for 10 h	44,000	100	100	100	100	100	100	50
CRI Sodium BP IV over 10 h 6,600 IU/kg bwt/h for 10 h	66,000	100	100	100	100	100	100	96

The target was fixed as *fT > MIC* equal to at least 40% of the dosing interval. Data were simulated for 500 horses receiving a dose of 22,000 IU BP/kg bwt per administration time, except for the two last simulations corresponding to an infusion of 44,000 IU/kg or 66,000 IU/kg for 10 h per day. The total daily BP dose is provided for each dosing regimen. Efficacy was expected when the PTA was higher than 90%.

The PTA with the different administration schemes by IV route demonstrated that a Constant Rate Infusion (CRI) of sodium BP for 10 h a day would be far more efficacious than IV boluses every 6 or 8 h. As an example, a CRI dose of 44,000 IU/kg bwt for 10 h would lead to PTA > 90% for MIC of 0.5 μg/mL, while higher daily doses of 66,000 or 88,000 IU/kg bwt delivered by IV boluses of 22,000 IU/ kg bwt every 8 h or 6 h, respectively, would not achieve the target of *fT > MIC* of 40% for MIC as low as 0.125 μg/mL.

By IM route, procaine BP alone q12h or q24h would lead to a PTA > 90% for an MIC of 0.25 μg/mL, while the same PTA would only be attained for MIC of 0.125 μg/mL and 0.064 μg/mL with procaine BP/benzathine BP combination q24h and penethamate hydriodide, respectively.

### 3.3 Local tolerance

The multiparametric pain score values were from 0 to 4/21, with a median of 0/21, showing a good tolerance to intramuscular injections.

For swelling score, median values were 0 [0–1] for procaine BP alone, 0 [0–1] for procaine BP/benzathine BP combination, and 0 [0–2] for penethamate hydriodide. ANOVA did not reveal any significant difference between treatments for this score.

For induration score, median values were 0 [0–1] for procaine BP alone, 0 [0–2] for procaine BP/benzathine BP combination, and 0.50 [0–2] for penethamate hydriodide. The induration score of penethamate hydriodide was significantly higher than procaine BP alone on the right side of the horse [median of 1 (0–2) and 0 (0–1), respectively; *p* = 0.045]; and induration score of procaine BP/benzathine BP combination was significantly higher than procaine BP alone on the left side [median of 0 (0–2) and 0 (0–1), respectively; *p* = 0.025]. Left gluteal muscles were used for Day 2 injection and penethamate hydriodide, and only 15 mL was injected per horse on that day, i.e., a volume lower than for procaine BP alone or procaine BP/benzathine BP combination (29.4 and 35.0 mL, respectively, for a 500-kg horse).

**Figure 2:**
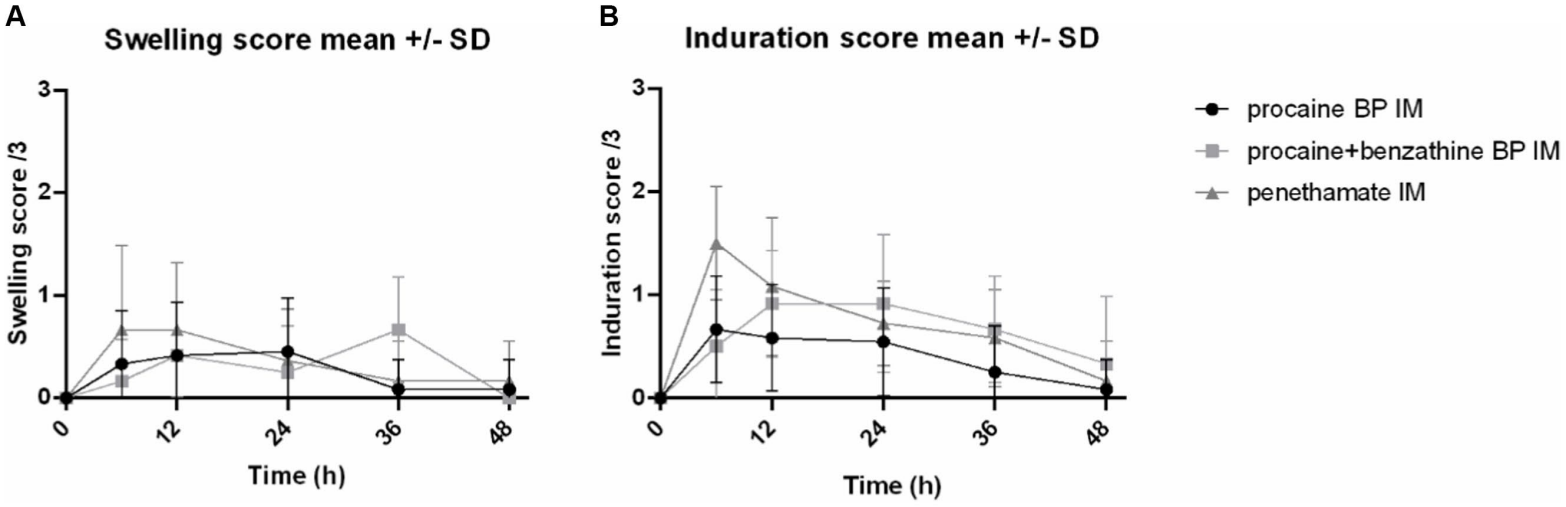
Swelling and induration scores of the injected sites. Representation of the swelling and induration scores at IM injection sites for 48 h after injection of three formulations: procaine BP (Dépocilline®, 17,800 IU/kg bwt BP), procaine BP/benzathine BP combination (Duplocilline®, 22,072 IU/kg bwt BP), and penethamate hydriodide (Penetavet®, 21,205 IU/kg bwt BP for a 500-kg horse). **(A)** Swelling scores. **(B)** Induration scores. Mean ± S.D. of left and right sides for the 6 horses.

From inspection of Figure 2, there is a tendency for penethamate hydriodide to elicit an early inflammation (swelling and induration) for 6 h after injection, whereas for procaine BP/benzathine BP combination, the reaction was delayed.

**Figure 3:**
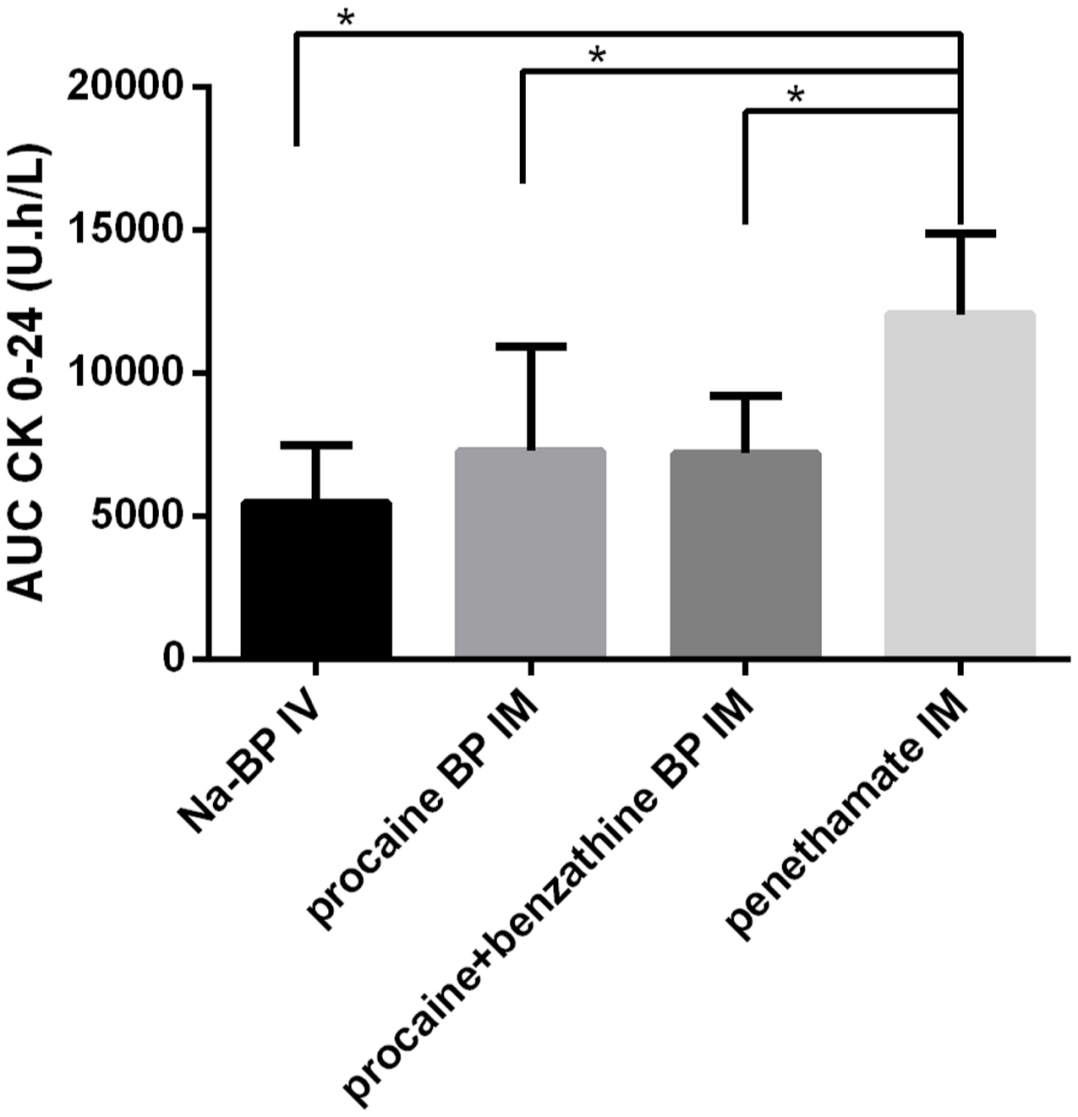
Area under the curve of plasmatic CK concentrations for 24 h following the first injection. AUC CK from 0 to 24 h is represented after injection with four different treatments: sodium BP IV, 22,000 IU/kg bwt BP, procaine BP (Dépocilline®, 17,800 IU/kg bwt BP), procaine BP/benzathine BP combination (Duplocilline®, 22,072 IU/kg bwt BP), and penethamate hydriodide (Penetavet®, 21,205 IU/kg bwt BP for a 500 kg horse). Mean ± S.D. for the six horses. *indicates a statistically significant difference.

Muscular lysis due to first injection was estimated by quantification of CK plasma concentrations over 24 h and the use of equations from Lefebvre et al. (13). Area under the curves of CK plasma concentrations from 0 to 24 h after the first injection was of 5,483 ± 2,003 U.h/L for sodium BP IV, 7,299 ± 3,638 U.h/L for procaine BP alone, 7,201 ± 2,025 U.h/L for procaine BP/benzathine BP combination, and 12,070 ± 2,838 U.h/L for penethamate hydriodide, with AUC after penethamate hydriodide significantly higher than the three other formulations (Figure 3). Estimated quantity of muscle loss for the first injection was 3.5 ± 8.9 g for procaine BP alone, 3.3 ± 4.5 g for procaine BP/benzathine BP combination, and 12.8 ± 5.1 g for penethamate hydriodide with injected volumes of 29.4, 35.0, and 30.0 mL for a 500-kg horse, respectively. With doses harmonized to 22,000 IU/kg bwt IM and by considering a muscle lysis proportional to the injected volume for each formulation, the muscle lysis would be of 4.3 ± 11.0 g for procaine BP alone, 3.3 ± 4.5 g for procaine BP/benzathine BP combination, and 13.3 ± 5.3 g for penethamate hydriodide with corresponding injected volumes of 36.5, 34.9, or 31.1 mL, respectively, for a 500-kg horse.

## 4 Discussion

Subtherapeutic antimicrobial dosing regimens not only increase the likelihood of poor clinical outcomes but also raise a higher risk of promoting antimicrobial resistance. Pharmacokinetics/pharmacodynamics approach can help optimize dosage regimens in humans (18) and animals (17).

The three IM formulations tested in this study are currently authorized in France for the same indications in horses. We administered them at dosing regimens in accordance with the SPC of each formulation. However, the three formulations led to very different PK profiles associated with varying bioavailabilities, with penethamate hydriodide and the procaine BP/ benzathine BP combination exhibiting lower bioavailabilities compared with procaine BP alone. One hypothesis could be a slower absorption rate of BP contained in formulations with penethamate hydriodide and the procaine BP/benzathine BP combination, potentially leading to local degradation of BP before reaching systemic circulation. Additionally, incomplete conversion of penethamate hydriodide into BP could contribute to the lower bioavailability observed for this formulation.

From the PK parameters calculated for these IM formulations but also with the IV formulation, we were able to simulate the PK profiles that would be obtained with dosage regimens different from those in the SPC and IV; we more specifically investigated the potential interest of a CRI. From simulated PK data, we then predicted efficacy of each formulation and each dosing regimen by conducting a PK/PD analysis. A recent study of our group, including BP PK data from other sources, showed that a *f*T > MIC of 40% could be achieved for MIC up to 0.25 μg/mL in 90% of horses with 22,000 IU/kg of IM procaine BP once a day (14). In the current study, we confirmed these data while providing PTA for different formulations and dosage regimens that could be used by practitioners. From a PK/PD perspective, we showed that an IV CRI at 66,000 IU/kg bwt/day of sodium BP for 10 h a day would be the most efficient treatment with BP, which able to reach a PTA > 90% against bacteria with an MIC up to 1 μg/mL. Procaine BP 22,000 IU/kg bwt q12h, as reported in textbooks, was the most efficient treatment by IM route, which able to reach a PTA > 90% with MIC up to 0.25 μg/mL and a PTA of 83% for MIC until 0.5 μg/mL. Regarding IM treatments with a constraint of q24h, procaine BP alone would be more efficacious than procaine BP/benzathine BP combination or penethamate hydriodide, despite daily administrations as high as 22,000 IU/kg, which is not supported by the SPC recommendations of the last two formulations.

The use of an IV CRI is an innovative and less frequently used method. Edwards et al. (5) already studied this mode of administration for BP and found that a loading dose of 2,314 IU/kg bwt (1.3 mg/kg) followed by a CRI of 4,450 IU/kg bwt/h (2.5 mg/kg/h) of sodium BP for 24 h should maintain total BP concentrations of 2 μg/mL corresponding to free BP concentrations of 0.8 μg/mL by considering protein binding of 60% in horses. Due to the very short half-life of BP, the steady-state will be quickly reached, and a loading dose is clearly not required (19). With the PK parameters and their variabilities estimated in our study and by considering BP protein binding of 60%, we estimated that a CRI of 6,600 IU/kg bwt/h for 10 h a day would likely result in a successful treatment for pathogens with an MIC of 1 μg/mL, making CRI a promising method of administration of sodium BP in the horse. By contrast, intermittent IV administration of boluses has resulted in far lower PTA even for MIC as low as 0.125 μg/mL due to the very short BP half-life, and this mode of administration should probably be discouraged. Furthermore, the total daily doses of sodium BP needed for CRI are equal or lower than daily doses used for IV boluses, an interest for both reducing environmental contamination risk, commensal flora modifications and being financially attracting. Restrictions to the CRI method are the infrastructure, personnel, and material needed that make its application difficult in the field. Indeed, BP has poor stability at ambient temperature, and solutions of sodium BP used for CRI should be stored at or below room temperature (20° to 25°C) and replaced every 12 h (5). In that purpose, using a frozen pack to put the infusion pack containing diluted sodium BP would be convenient, but the cost of an infusion pump and the necessary handling can probably limit this mode of administration to hospital use.

The adverse reaction shown by a mare using penethamate hydriodide was similar to reactions occasionally observed in clinical settings using procaine BP, which was linked to procaine (a local anesthetic) toxicity (20). Interestingly, penethamate hydriodide also possesses some local anesthetic activities due to the diethylaminoethanol ester, but we cannot conclude on the exact cause of the reaction (21). Overall, tolerance to the injections was good with dosage regimen and intervals used in the present study. Gordon et al. (22) have studied the effects of an administration of 22,000 IU/kg bwt of procaine BP q12h for 5 days on six horses in the caudal cervical muscles, and they found moderate but significantly elevated values of Serum Amyloid A and fibrinogen from their baselines in 3 of 6 horses at the end of administrations and also observed mild to moderate swelling at injection sites from day 2 to day 12 in all horses. In our study, estimated quantity of muscle damaged from the first IM injections ranged from 3 to 13 g for a horse of 500 kg and could be considered as negligible. Procaine BP/benzathine BP combination and penethamate hydriodide led to increased induration at the injection site; and penethamate hydriodide led to significantly higher muscular lysis compared with the other IM-tested drugs. However, injected volumes in our study were slightly higher on the first day of procaine BP/benzathine BP combination compared with procaine BP alone or penethamate hydriodide (35.0 mL, 29.4 mL, and 30.0 mL for a 500-kg horse, respectively), and the amount of BP was not the same and was slightly higher with procaine BP/benzathine BP combination (22,072 IU/kg bwt) and penethamate hydriodide (21,205 IU/kg bwt) than with procaine BP alone (17,800 IU/kg bwt).

The main limitation of our study is that we indirectly evaluated the effects of the different formulations of BP with a PK/PD index (ft > MIC) which is only a surrogate of clinical effectiveness (23). Another limitation is that the effect of the BP formulations cannot be distinguished from the effect of the medicines (including excipients) themselves, and that, we did not explore repeated administration effect on tolerance. The site of injection has been demonstrated to influence PK parameters of BP (7), and it cannot be excluded that the tolerance could also depend on the site of injection. Whatever the formulation, tolerance of horses to repeated administrations can be improved by alternating injection sites (i.e., neck, thigh, and gluteal muscles) and good injection technique. Some manufacturers also recommend a maximum volume of intramuscular injection of 20 mL per point of injection.

Overall, procaine BP alone seems the best option for treatment with BP by IM route. For pathogens with MIC up to 0.25 μg/mL, one daily IM injection of 22,000 IU/kg bwt of procaine BP could be enough, thus potentially increasing tolerance and improving observance. Over 230 strains of *S. equi* from the EUCAST database, only three strains had MIC higher than 0.016 μg/mL (24), suggesting that one daily intramuscular administration of procaine BP alone, procaine BP/benzathine BP combination, or penethamate hydriodide at 22,000 IU BP/kg bwt should be adequate for this bacterial species. For other pathogens, 22,000 IU/kg of procaine BP once or twice a day, as recommended in textbooks, could be proposed to achieve an appropriate efficacy against pathogens with MIC up to 0.25 μg/mL, but a good practice would be to determine precisely the MIC to decide the appropriateness of BP treatment.

In conclusion, all the formulations tested in this study should be adequate to treat infections with susceptible *S. equi,* while the procaine BP by IM route and CRI for 10 h a day by IV route would be the most efficient on less susceptible bacteria. Our study did not explore the optimal duration of therapy because PK/PD consideration cannot document this point (25), and further investigation in this regard is essential. Shortening the therapy duration could potentially enhance compliance and increase tolerance. Further studies are also required to check if the targeted free BP concentrations are achieved using the proposed dosing regimens in diseased animals as the health status may influence pharmacokinetic parameters (26).

## Data availability statement

The raw data supporting the conclusions of this article will be made available by the authors, without undue reservation.

## Ethics statement

The animal study was approved by the Comité d’éthique de Pharmacologie-toxicologie de Toulouse-Midi Pyrénées. The study was conducted in accordance with the local legislation and institutional requirements.

## Author contributions

AF: Conceptualization, Formal analysis, Investigation, Software, Validation, Writing – original draft, Writing – review & editing. BR: Data curation, Formal analysis, Investigation, Software, Validation, Writing – review & editing. LC: Data curation, Formal analysis, Investigation, Validation, Writing – review & editing. TK: Formal analysis, Investigation, Validation, Writing – review & editing. ML: Data curation, Formal analysis, Validation, Writing – review & editing. P-LT: Formal analysis, Methodology, Validation, Writing – review & editing. AB-M: Conceptualization, Formal analysis, Methodology, Supervision, Validation, Writing – review & editing. EL: Conceptualization, Data curation, Formal analysis, Funding acquisition, Investigation, Methodology, Project administration, Resources, Software, Supervision, Validation, Visualization, Writing – original draft, Writing – review & editing.
