# Supplementary material for: Predicted efficacy and tolerance of different dosage regimens of benzylpenicillin in horses based on a pharmacokinetic study with three IM formulations and one IV formulation
Source: Front Vet Sci. 2024 May 31;11:1409266. doi: 10.3389/fvets.2024.1409266 (PMC11176610; doi:10.3389/fvets.2024.1409266)
Supplement: Supplementary file 1 [file Data_Sheet_1.docx]

Supplementary Material

# Table S1: swelling score.

This injection score is from 0 to 3, 0 is a normal injection site, 3 being the most swollen. Injection site was evaluated before injection, at 6h and 12h after injection, then daily until day6.

| Score | Criteria |
| --- | --- |
| 0 | No swelling |
| 1 | Mild swelling (diameter ≤ 5 cm) |
| 2 | Moderate swelling (5 < diameter ≤ 10 cm) |
| 3 | Severe swelling (diameter > 10 cm) |

# Ta**ble S2: induration score**.

This injection score is from 0 to 3, 0 is a normal injection site, 3 is the worst. Injection site was evaluated before injection, at 6h and 12h after injection, then daily until day6.

| Score | Criteria |
| --- | --- |
| 0 | No induration |
| 1 | Mild induration (diameter ≤ 2 cm) |
| 2 | Moderate Induration (2 < diameter ≤ 8 cm) |
| 3 | Severe induration sévère (diameter > 8 cm) |

# Table S3: global pain score.

This global pain score is from 0 to 21, 21 being the worst. This score was adapted from de Grauw and Van Loon 2016 and Gleerup 2015.

| **Physiological data** | **Criteria** | **Score** |
| --- | --- | --- |
| Heart rate | Normal compared to initial value (<10% increase)  11–30% increase  31–50% increase  >50% increase | 0  1  2  3 |
| Respiratory rate | Normal compared to initial value (<10% increase)  11–30% increase  31–50% increase  >50% increase | 0  1  2  3 |
| Digestive sounds (bowel movement) | Normal motility  Decreased motility  No motility - Hypermotility | 0  1  2 |
| Response to palpation of the painful area | No reaction to palpation  Mild reaction to palpation  Resistance to palpation  Violent reaction to palpation | 0  1  2  3 |
| Equine pain face (from Gleerup 2015) | Facial expression of a pain free, relaxed and attentive horse, ears forward  Facial expression of a horse in pain, assymetrical ears  Facial expression of a horse in pain, ears facing back | 0.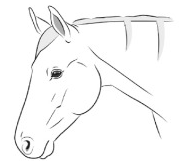  1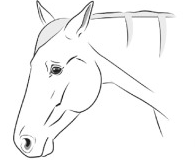  2 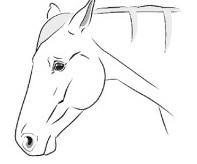 |
| Posture (weight distribution, comfort) | Stands quietly, normal walk  Occasional weight shift, slight muscle tremors  Non-weight bearing, abnormal weight distribution  Analgesic posture (attempts to urinate, prostration, muscle tremors) | 0  1  2  3 |
| Interactive behaviour | Pays attention to people  Exaggerated response to auditory stimulus  Excessive-to-aggressive response to auditory stimulus  Stupor, prostration, no response to auditory stimulus | 0  1  2  3 |
| Lameness | Lameness grading according to AAEP /5   - 0 = no lameness perceptible under any circumstances - 1 = lameness is difficult to observe and is not consistently apparent, regardless of circumstances (eg, under saddle, circling, inclines, hard surface) - 2 = lameness is difficult to observe when walking or when trotting in a straight line but consistently apparent under certain circumstances (eg, weight carrying, circling, inclines, hard surface) - 3 = lameness is consistently observable at a trot under all circumstances - 4 = lameness is obvious at a walk - 5 = lameness produces minimal weight bearing in motion or at rest or a complete inability to move. | 0 no difference with initial exam T0  1 increase of one grade from T0  2 increase of more than 1 grade from T0 |
| TOTAL |  | /21 |

DE GRAUW, J.C. et VAN LOON, J.P.A.M., 2016. Systematic pain assessment in horses. In : The Veterinary Journal. Vol. 209, pp. 14 22. DOI 10.1016/j.tvjl.2015.07.030.

GLEERUP, Karina B, FORKMAN, Björn, LINDEGAARD, Casper et ANDERSEN, Pia H, 2015. An equine pain face. In : Veterinary Anaesthesia and Analgesia. 2015. Vol. 42, n° 1, pp. 103 114. DOI 10.1111/vaa.12212
